# Hydrogen peroxide yields mechanistic insights into human mRNA capping enzyme function

**DOI:** 10.1371/journal.pone.0186423

**Published:** 2017-10-13

**Authors:** Nicholas J. Mullen, David H. Price

**Affiliations:** Department of Biochemistry, University of Iowa, Iowa City, Iowa, United States of America; George Mason University, UNITED STATES

## Abstract

Capping of nascent RNA polymerase II (Pol II) transcripts is required for gene expression and the first two steps are catalyzed by separate 5′ triphosphatase and guanylyltransferase activities of the human capping enzyme (HCE). The cap is added co-transcriptionally, but how the two activities are coordinated is unclear. Our previous in vitro work has suggested that an unidentified factor modulates the minimum length at which nascent transcripts can be capped. Using the same well-established in vitro system with hydrogen peroxide as a capping inhibitor, we show that this unidentified factor targets the guanylyltransferase activity of HCE. We also uncover the mechanism of HCE inhibition by hydrogen peroxide, and by using mass spectrometry demonstrate that the active site cysteine residue of the HCE triphosphatase domain becomes oxidized. Using recombinant proteins for the two separated HCE domains, we provide evidence that the triphosphatase normally acts on transcripts shorter than can be acted upon by the guanylyltransferase. Our further characterization of the capping reaction dependence on transcript length and its interaction with the unidentified modulator of capping raises the interesting possibility that the capping reaction could be regulated.

## Introduction

The addition of the m7GpppN cap is the first step in the biochemical processing of nascent mRNAs, and it is required for eukaryotic gene expression [1–3]. It protects the 5′ end of mRNA from degradation to ensure stability [4], is involved in nuclear export, and is required for efficient pre-mRNA splicing [5, 6] and translation [7]. There are three successive enzymatic activities involved in the biosynthesis of a mature cap: the RNA triphosphatase, the guanylyltransferase, and the mRNA cap guanine-N7 methyltransferase [8]. The RNA triphosphatase activity first removes the gamma phosphate from the 5′ end of a nascent triphosphate RNA, yielding a diphosphate RNA. The guanylyltransferase activity then transfers a GMP moiety from GTP to the diphosphate RNA through a covalent GMP-enzyme intermediate, resulting in a 5′-5′ triphosphate linkage (the combination of these two reactions is hereafter referred to as “capping”). Finally, the cap methyltransferase methylates N7 of the guanosine, yielding the mature cap. In yeast, these three activities are encoded on individual polypeptides [8], while in metazoans the triphosphatase and guanylyltransferase activities make up distinct domains of a single polypeptide [9], which in humans is called the human capping enzyme (HCE).

Capping occurs co-transcriptionally [10, 11], and is functionally coupled to Pol II transcription [12]. Capping is thought to occur as soon as the nascent transcript emerges from the Pol II RNA exit channel. Nascent RNA reaches the surface of yeast Pol II at a length of about 15 nt [13, 14], but previous studies have found that human RNA remains uncapped until the nascent transcript reaches a length of at least 20 nt [10]. HCE has been shown to interact with the hSpt5 subunit of the DRB-sensitivity inducing factor (DSIF), which is crucial for the transition of the Pol II elongation complex from the promoter proximal paused state to productive elongation [15, 16]. It was also recently reported that oncogenic deregulation of c-Myc increases HCE association with Pol II at c-Myc-controlled genes, and that c-Myc overexpression relies on increased HCE activity [17].

In both yeast and mammals, the capping apparatus has been extensively characterized, and there are several key structural, functional, and mechanistic differences between the two taxa [8]. In yeast, the RNA triphosphatase (Cet1) and the guanylyltransferase (Ceg1) make up a complex consisting of a Cet1 homodimer and either one or two interacting Ceg1 molecules [18]. A recent cryo-EM study supported the interaction of a Ceg1-Cet1-Cet1-Ceg1 heterotetramer with the Pol II elongation complex [19]. In this structure, Cet1 interfaced with the RNA exit channel of Pol II, and this interaction was greatly enhanced by the presence of a nascent triphosphate RNA [19]. It is not clear if HCE shares the requirement for triphosphatase dimerization, but it seems unlikely, since mammalian triphosphatase domain uses a different mechanism than Cet1 and bears no structural similarities [20]. Both Ceg1 and the mammalian capping enzyme guanylyltransferase domain have been shown to interact with the Ser5 phosphorylated C-terminal domain (CTD) of the large subunit of Pol II. However, the CTD interaction sites on the yeast and human enzymes are structurally and chemically distinct [21]. In the absence of Cet1, the phosphorylated CTD inhibits charging of Ceg1 (Ceg1-GMP intermediate formation), and this inhibition is relieved by Cet1 [22]. Because the mammalian triphosphatase and guanylyltransferase domains are covalently linked, it is not clear if the phosphorylated CTD has a similar influence on the capping reaction in humans.

Because of the central importance of capping in gene expression, we have sought to characterize the interaction of HCE with elongating Pol II and the transcript length dependence of the capping reaction in vitro. We recently showed that the optimal transcript length for capping to occur in our in vitro system is shorter after a high salt wash, which removes all accessory factors and leaves only engaged Pol II on the immobilized template and the nascent RNA [23]. Furthermore, after a low salt wash that leaves many factors still associated with the elongation complex, the transcript length for efficient capping was not lowered. Taken together, these results suggest that there is an unknown factor that normally associates with the early Pol II elongation complex and inhibits capping at the earliest possible point. This same study also showed that treatment with hydrogen peroxide (H_2_O_2_) inhibits capping in the complete system (without any wash), but does not inhibit capping when HCE is added back to high salt washed elongations complexes. This suggested that HCE was not the target of H_2_O_2_ that leads to a loss of capping, and it also raised the question of whether the unidentified wash-sensitive inhibitor of capping might be involved. Because of these facts, we endeavored to identify the target of H_2_O_2_.

## Materials and methods

### Protein purifications

Recombinant human capping enzyme was purified as described previously (Moteki and Price, 2002). Recombinant truncated human capping enzyme guanylyltransferase domain (GTase) and triphosphatase domain (TPase) were cloned into a pET21a plasmid, with an N- and C-terminal His tag, respectively. His-TPase-pET21a contains residues 1–215 of HCE with an N-terminal His tag. pET21a-GTase-His includes residues 216–597 of HCE and a C-terminal His tag. The truncation point was chosen based on sequence conservation between human, mouse, and frog; in frog there is a G insertion between human/mouse S215 and A216. Each plasmid was expressed in *E*. *coli* DE3 cells. TPase was purified with Ni-NTA followed by Mono Q; GTase was purified with Ni-NTA followed by Mono S.

### In vitro transcription assays

Preinitiation complex (PIC) formation, limiting [α-^32^P]CTP pulse, and early elongation complex (EEC) isolation was described previously [24]. PIC formation was accomplished by 30 min incubation of HeLa nuclear extract (HNE) and an immobilized CMV promoter-containing DNA template in the presence of 20 mM HEPES pH 7.6, 60 mM potassium acetate, 5 mM magnesium acetate, 0.5 U/L SUPERase·In, and varying concentrations of either dithiothreitol (DTT) or H_2_O_2_. Initiation was accomplished with a 30 s pulse with 500 μM ATP, UTP, and GTP with limiting [α-^32^P]CTP. If EECs were to be isolated, the reaction was stopped with a solution containing 20 mM EDTA (to chelate the magnesium), 20 mM HEPES pH 7.6, 0.2 mg/ml BSA, 0.02% Tween20, and either 60 mM (low salt) or 1.6 M (high salt) potassium acetate. The stop was followed by either a low salt wash (LSW) containing 60 mM potassium acetate or high salt wash (HSW) containing 1.6 M potassium acetate in the same solution just described. If no EECs were to be isolated, the reaction was stopped with a solution containing 100 mM Tris pH 7.6, 1% Sarkosyl, 20 mM EDTA, and 0.2 mg/ml Torula yeast RNA (Sigma, R6625). The labeled RNA was then isolated by phenol extraction and precipitated in 0.5 M ammonium acetate and 95% ethanol. The resulting pellet was then washed with 70% ethanol and resuspended in RNA loading buffer containing 9.35 M Urea, 0.25x TBE with 0.0075% bromophenol blue and 0.03% xylene cyanol. The resuspended samples were then run on a 12% polyacrylamide denaturing gel (6 M urea) at 18 W for 70 min. Gels were dried and exposed overnight to an imaging plate and visualized by phosphorimaging (Fuji FLA-7000). The transcript lengths assigned to the different bands were based the site of initiation being the central A in the sequence GTC**A**GAT which we determined by sequencing nascent HCMV transcripts. This represents a revision of the transcription start site we used recently [23] which was based on earlier estimates [25, 26].

All experiments were performed at least twice in exact duplicate assays or assays that were highly similar. Significance was also achieved by carrying out time courses and titrations demonstrating graded effects.

### RNA reisolation and naked RNA enzymatic addback assay

Pulsed PIC reactions were stopped with a buffer containing 20 mM HEPES pH 7.6, 60 mM potassium acetate, 0.2 mg/ml BSA, 20 mM EDTA and 1% Sarkosyl. 60 μg of glycogen was then added to allow pellet formation, and the RNA was phenol extracted as described previously. After resuspension in a buffer containing 20 mM HEPES, 60 mM potassium acetate, 0.2 mg/ml BSA, and 0.5 U/L SUPERase·In, reisolated RNA was incubated with addback solution at a final magnesium acetate concentration of 5 mM at 37°C for 10 min.

### Mass spectrometry analysis

20 μg of HCE was incubated at 0.6 mM H_2_O_2_ or water as a control for 10 min before being quenched with 6 mM DTT, in a solution containing 20 mM HEPES (pH 7.6), 250 mM potassium acetate, 5 mM magnesium acetate, and subjected to mass spectrometry by the Proteomics Core facility at the University of Iowa. Desalted samples were digested overnight with Promega Gold sequencing grade Trypsin in 25 mM ammonium bicarbonate at a 1:50 ratio. Desalted peptides were dehydrated in a Thermo speed vacuum and re-suspended in 3% Acetonitrile (LCMS grade) and 0.1% formic acid which constitutes mobile phase A. Mobile phase B consists of 90% acetonitrile and 0.1% formic acid. 3 uL of each sample was injected on a hand-packed column of 75 um id x 10 cm length using a Dionex Ultimate 3000 UHP nanoLC. A 10 port valve on the Ultimate holds both a sample loop and a New Objective desalting cartridge. Linear gradients flow at 300 nL/min and pass from 3 to 45% B over 60 minutes. Flow is directed toward a Thermo LTQ/XL linear ion trap where the spray voltage is maintained between 1600 and 1700 V. Data are collected with a survey scan in the mass region of 300 to 1400 Th followed by isolation (Q = .25) and fragmentation (NCE = 30) of the top six most abundant ions. Collected data was interpreted with the aid of the 2.5 release of the MASCOT search engine running a human database of 178,749 sequences paired to a decoy made of reversed sequences. Protein identifications were restricted to <1% FDR.

## Results and discussion

### H_2_O_2_ treatment of PICs leads to rapid and mostly irreversible loss of capping

An in vitro transcription system with HeLa nuclear extract (HNE) and CMV promoter-containing DNA template was used to assess the effect of H_2_O_2_ on capping of nascent transcripts [23]. Preinitiation complexes (PICs) were formed by 30 minute incubation of HNE and template DNA, and reactions were pulsed for 30 seconds in the presence of 500 μM ATP, UTP, GTP and limiting [α-^32^P]CTP. Limiting CTP during the pulse causes Pol II to stall at each spot where a C is the next base to be added. This leads to the formation of discrete bands of different transcript lengths (G13, G18, U22, A28). Note that these designations are 3 nt shorter than used earlier [23] due to reassignment of the transcription start site (see Material and methods). As demonstrated earlier comparing transcripts generated with HNE before and after depletion of the capping enzyme [27], addition of a 5′ cap lowers the mobility to the equivalent of 1.5–2 additional nucleotides (1 nucleoside added and one charge removed). Thus, for each of these transcript lengths, the efficiency of capping can be assessed by comparing the intensities of the upper and lower bands, corresponding to the capped and uncapped transcripts, respectively. We have shown previously that treatment of PICs with H_2_O_2_ for 30 min before initiation results in a loss of capping through an unknown mechanism [23].

**Fig 1:**
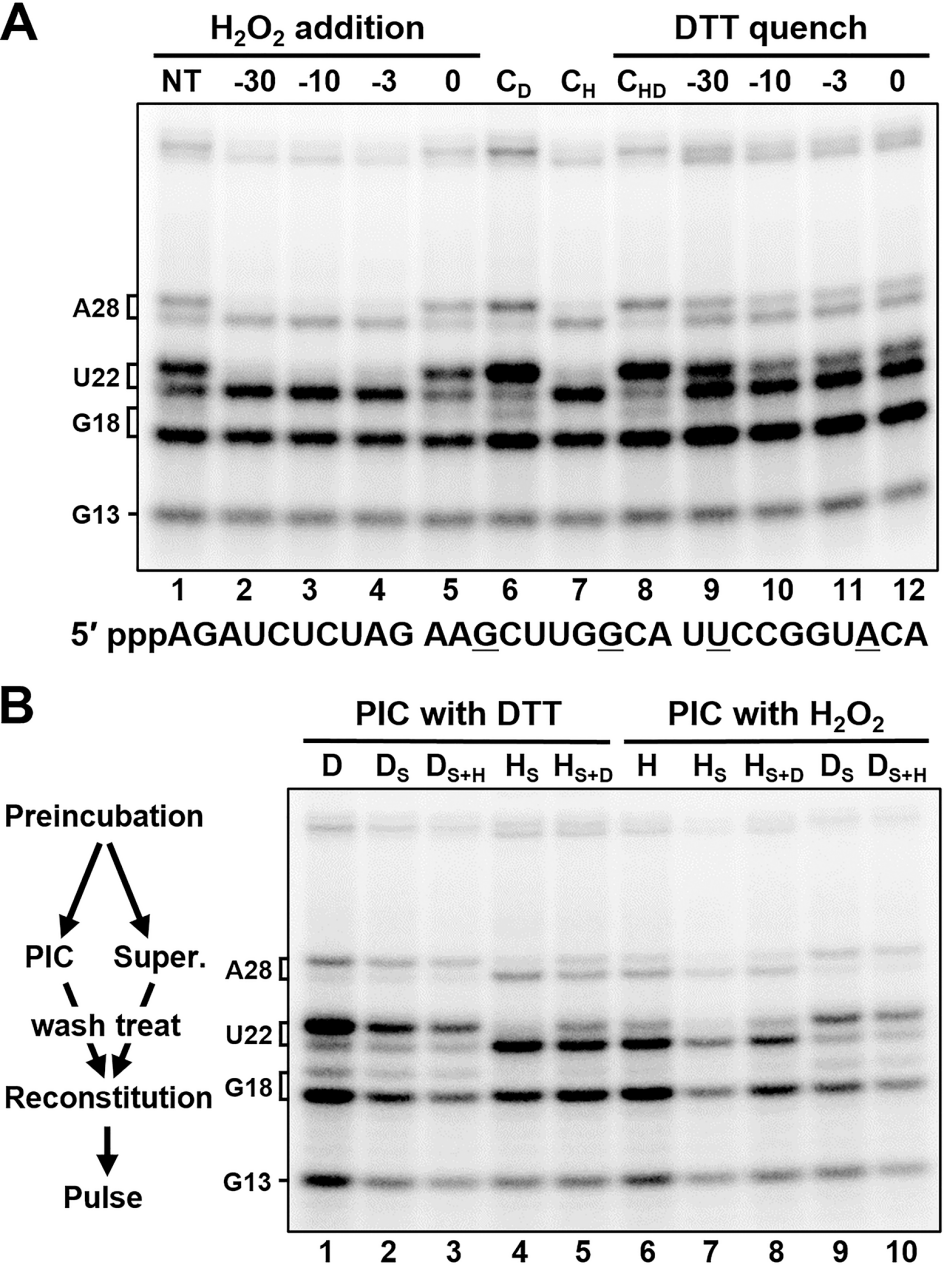
H_2_O_2_ rapidly and irreversibly inactivates its target, which is not associated with the PIC. All H_2_O_2_ treatments were to a final concentration of 0.6 mM. All DTT treatments were to 6 mM (10-fold excess over H_2_O_2_). *A*, PICs were formed for at least 30 min and pulsed at time 0. H_2_O_2_ addition: H_2_O_2_ added at indicated time points; or no treatment (NT). C_D_: DTT present initially. C_H_: H_2_O_2_ present initially. C_HD_: H_2_O_2_ premixed with DTT, present initially. DTT quench: H_2_O_2_ present initially, quenched with 10-fold molar excess of DTT at indicated time points. *B*, PICs with indicated treatment were formed for 30 min, after which their supernatants were removed, remaining PICs were low salt washed, and indicated supernatants were added back to washed PICs after optional treatment of supernatant with the opposite compound. Lanes 1 and 6 were DTT (D) or H_2_O_2_ (H) treated controls that did not undergo supernatant separation. D_S_ = supernatant from DTT treated PICs; D_S+H_ = D_S_ treated with H_2_O_2_; H_S_ = supernatant from H_2_O_2_ treated PICs; H_S+D_ = H_S_ treated with DTT.

An experiment was designed to examine the kinetics of the H_2_O_2_-dependent loss of capping and determine if the inhibition was reversible. The control reaction generated uncapped transcripts ending with G13 and G18 and mostly capped U22 and A28 (Fig 1A, lane 1). H_2_O_2_ (0.6 mM final) was added to bring the 30 min PIC formation reaction 30, 10, 3, or 0 min before the pulse. Analysis of the transcripts generated during the 30 s pulse showed that H_2_O_2_ did not inhibit initiation, but induced loss of capping that was complete after just 3 minutes of treatment (Fig 1A, lane 4). The effect was not instantaneous because H_2_O_2_ had virtually no effect when added with the pulse (Fig 1A, lane 5). Inclusion of 6 mM DTT during the PIC formation had a positive effect on initiation and capping (Fig 1A, lane 6). When H_2_O_2_ was pre-quenched with a 6 mM DTT, no inhibition of capping was observed (Fig 1A, lane 8). To determine if the inhibition of capping by H_2_O_2_ was reversible, H_2_O_2_ was added at the beginning of the PIC formation reaction (-30 min) and DTT added at different times. When the DTT was added immediately after the H_2_O_2_ (before oxidation was complete) most of the capping activity was retained (Fig 1A, lane 9). At all other times, capping was still mostly inhibited (Fig 1A, lanes 10–12). The fraction that is reversible can be rescued almost instantly, since a quench 10 minutes before initiation had the same capping output as a quench simultaneous with initiation (compare lanes 10 and 12). We conclude that H_2_O_2_ acts rapidly to inhibit capping and makes a modification that is mostly irreversible, even in large excess of a strong reducing agent. The fact that H_2_O_2_-dependent loss of capping is mostly irreversible allows us to directly search for the H_2_O_2_ target in nuclear extracts by oxidizing and quenching the PICs and then adding unoxidized factors back to the reaction and look for a rescue of the capping defect.

We first decided to determine if the target was stably associated with the PIC or soluble. To accomplish this, an assay was designed that would allow the supernatant from one PIC-formation reaction to be added to the isolated PICs of another reaction. PICs were formed on an immobilized DNA template either in the presence of 6 mM DTT or 0.6 mM H_2_O_2_; the supernatants were then removed (or not in the case of the two controls) and the remaining PICs isolated by a low salt wash (60 mM potassium acetate) which removes soluble material. The supernatants were then optionally treated with the opposite compound and then recombined with each PIC in every possible permutation (Fig 1B). When the supernatant from the H_2_O_2_-containing PIC-formation reaction was added back to a PIC formed in the presence of DTT and then pulsed, capping was not observed (lane 4). Conversely, when the supernatant from the DTT-containing PIC-forming reaction was added back to a PIC formed in the presence of H_2_O_2_ and then pulsed, capping was observed (lane 9). Taken together, these two results demonstrate that the H_2_O_2_ target is not stably associated with the PIC.

### H_2_O_2_-dependent loss of capping is due to oxidation of catalytic residue in HCE triphosphatase domain

**Fig 2:**
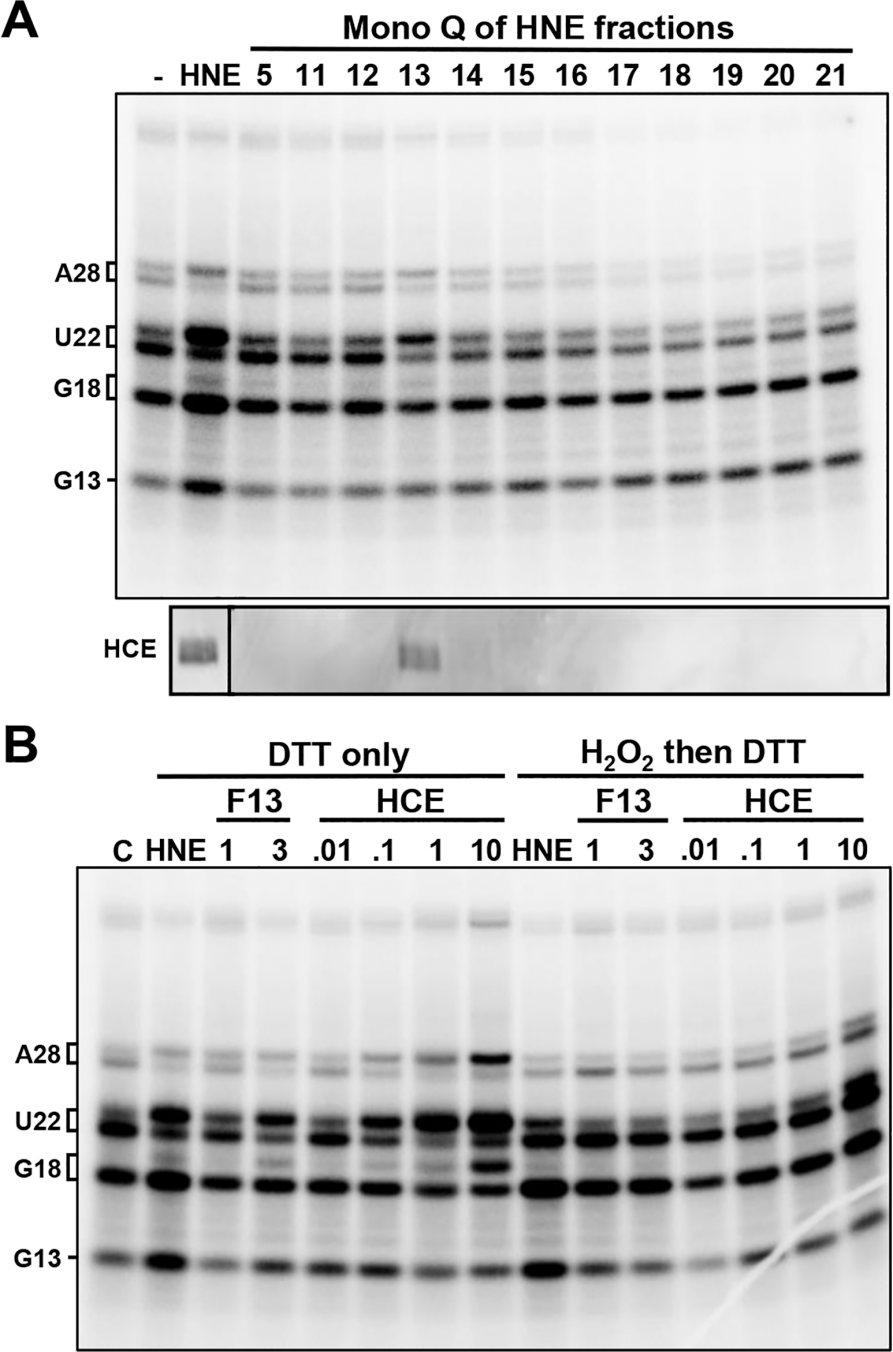
HCE is the target of H_2_O_2_. All PICs were treated with 0.6 mM H_2_O_2_ for 10 min, then quenched with 6 mM DTT. Indicated addbacks occurred after the quench and 5 min before the pulse. *A*, Addback of either HeLa nuclear extract (HNE) or a Mono Q fraction of HNE. Bottom panel shows Western blot for HCE in indicated fractions. *B*, Addback of HNE, Mono Q fraction 13 or indicated amounts of HCE (pmol).

Since the target of H_2_O_2_ was found in the supernatant, this allowed us to search for a factor that would rescue the capping defect using an add-back assay. Fractions from the chromatography of HNE on Mono Q were added back to reactions that were first treated with H_2_O_2_ and then quenched with DTT. As expected, add-back of HNE rescued the capping defect (Fig 2A, HNE). Of the Mono Q fractions, only fraction 13 rescued the capping defect. Surprisingly, a Western blot demonstrated that fraction 13 contained HCE (Fig 2A, bottom). To determine if HCE was actually the target, HNE or increasing amounts of fraction 13 or purified HCE were added back to oxidized/quenched reactions. All three were able to rescue the capping defect and both fraction 13 and HCE did so in a dose dependent manner (Fig 2B). The rescue was blocked by H_2_O_2_/DTT treatment of the material added back. We conclude that contrary to our earlier prediction [23], HCE is the target of H_2_O_2_ that leads to loss of capping. This discrepancy will be explained below.

**Fig 3:**
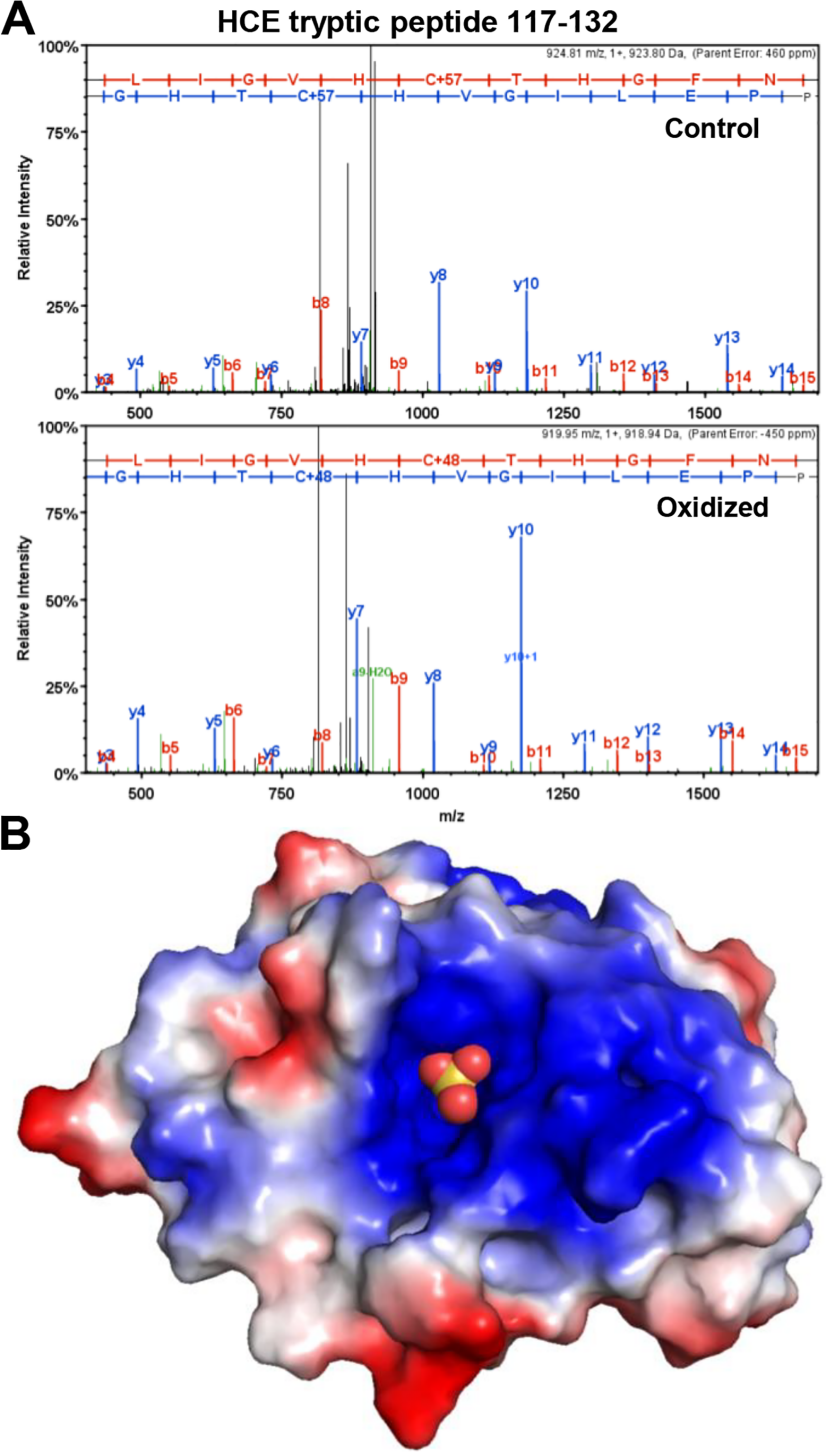
Cys126 is the site of oxidation in HCE. *A*, Spectra from MS/MS fragmentation analysis of tryptic peptide (aa 117–132) from HCE treated with DTT (Control) or 0.6 mM H_2_O_2_ and then quenched with 10-fold molar excess DTT (Oxidized). *B*, Model of HCE triphosphatase domain with active site Cys126 oxidized to sulfonic acid (balls). Model was built in PyMol using mouse triphosphatase domain oxidized with tungstate treatment (sulfonic acid group added) PDB 1I9T [20]. Positive (blue) and negative (red) charged regions are indicated.

To identify the site(s) of oxidation, mass spectrometry of HCE that had been treated with H_2_O_2_ or not was performed under the standard protocol of DTT reduction, cysteine alkylation with chloracetamide (+57) and overnight digestion with trypsin. Alkylation to carbamidomethyl-cysteine increases the residue mass to 160 Da. Cysteine residues oxidized to sulfonic acid are resistant to alkylation, and the addition of 3 oxygen atoms increases the residue mass to 151. Spectra (Fig 3A) and the associated fragment analyses (Table 1) from MS/MS fragmentation of the tryptic peptide (aa 117–132) revealed that in the control sample the sulfur atom of Cys126 was alkylated prior to digestion and the gap between C-terminal fragments y6 and y7 was 160, as expected. The same gap between the y6 and y7 fragments was only 151 Da in the peptide from oxidized HCE. This is consistent with tri-oxidation at Cys126 (103 Da Cys + 48 Da O3) due to H_2_O_2_ treatment of HCE prior to digestion. Cys126 is the catalytic residue in the active site of the HCE triphosphatase [28, 29], suggesting an obvious mechanism for HCE inhibition through inactivation of the triphosphatase. Sulfonic acid is the most oxidized state for a cysteine sulfur atom, and the oxidation reaction leading to its formation is not reversible by DTT [30]. Lower oxidation states are reversible by DTT and a small fraction of unoxidized Cys126 was observed in the oxidized sample that was treated with DTT before analysis. This explains the partial reversibility of inhibition of capping seen in our assays (see Fig 1A). We suggest that the highly basic environment of the active site (Fig 3B) helps to stabilize the highly oxidized residue, and this may explain why other sulfur-containing residues were not significantly modified.

**Table 1:** Fragment analysis of tryptic peptide 117–132 from control and H_2_O_2_ treated HCE.

		Control					Oxidized		
	B Ions		Y Ions			B Ions		Y Ions	
1	-	**N**	-	16	1	-	**N**	-	16
2	-	**P**	-	15	2	-	**P**	-	15
3		**P**	1,636.80	14	3	-	**P**	1,627.76	14
4	438.20	**E**	1,539.75	13	4	438.20	**E**	1,530.71	13
5	551.28	**L**	1,410.71	12	5	551.28	**L**	1,401.67	12
6	664.37	**I**	1,297.62	11	6	664.37	**I**	1,288.59	11
7	721.39	**G**	1,184.54	10	7	721.39	**G**	1,175.50	10
8	820.46	**V**	1,127.52	9	8	820.46	**V**	1,118.48	9
9	957.52	**H**	1,028.45	8	9	957.52	**H**	1,019.41	8
10	1,117.55	**C+57**	891.39	7	10	1,108.51	**C+48**	882.35	7
11	1,218.59	**T**	731.36	6	11	1,209.56	**T**	731.36	6
12	1,355.65	**H**	630.31	5	12	1,346.62	**H**	630.31	5
13	1,412.67	**G**	493.25	4	13	1,403.64	**G**	493.25	4
14	1,559.74	**F**	436.23	3	14	1,550.71	**F**	436.23	3
15	1,673.79	**N**	-	2	15	1,664.75	**N**	289.16	2
16	-	**R**	-	1	16	-	**R**	-	1

Fragments identified by MS/MS for B and Y ions are shown. See Materials and methods for details.

### The two HCE domains act independently, and the inhibitor of capping at G18 affects only the guanylyltransferase domain

**Fig 4:**
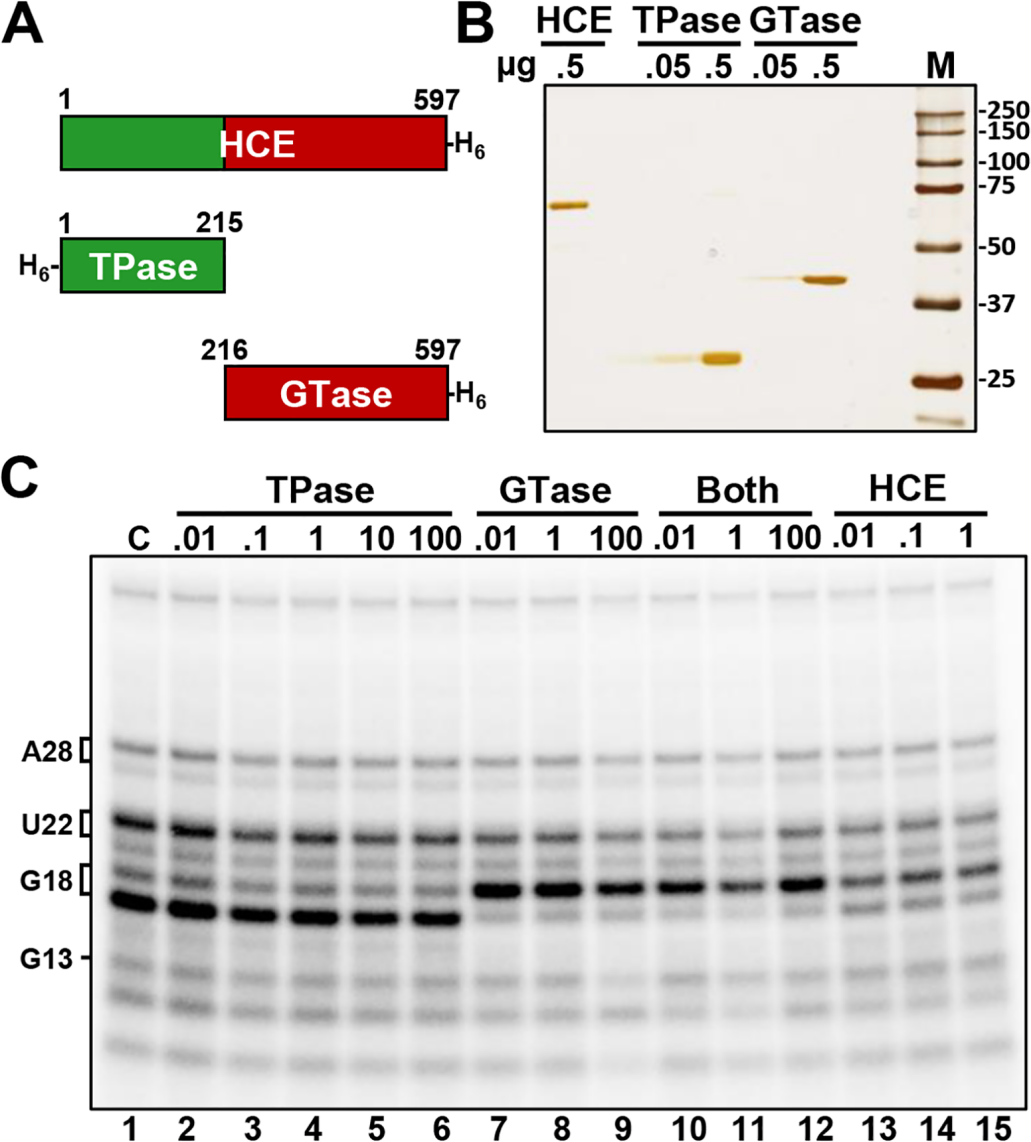
G18 in isolated elongation complexes is guanlylyated by HCE and the GTase alone. *A*, Schematic of recombinant human capping enzyme (HCE), recombinant triphosphatase domain (TPase), and recombinant guanalyltransferase domain (GTase). *B*, Silver stained SDS PAGE analysis of the indicated amounts of purified proteins. *C*, Addback of increasing amounts (pmol) of TPase, GTase, both TPase and GTase, or HCE to high salt washed elongation complexes.

To determine if the triphosphatase (TPase) and the guanylyltransferase (GTase) domains functionally interacted, we expressed and purified the two domains individually (Fig 4A and 4B). The activity of the TPase, GTase and a mixture of both was compared to the intact HCE. The proteins were incubated with high salt washed EECs (HSW EECs) in the presence of GTP for 3 minutes before the resulting RNAs were examined (Fig 4C). Compared to the control, in which no proteins were added, the TPase caused no mobility shift on G18 (lanes 2–6) and as expected, the combination of TPase and GTase (lanes 10–12) or intact HCE (lanes 13–15) caused the shift in mobility signifying addition of the cap. Unexpectedly, the GTase alone was able to efficiently guanylylate uncapped G18 (lanes 7–9). This was extremely surprising, since uncapped G18 in HSW ECs was presumed have a triphosphate end, but the fact that the GTase was able to cap it suggested that it instead exists as a diphosphate, meaning that the TPase had already acted on the elongation complex during the pulse, before isolation of the EECs. This suggests that the previously described unknown factor that down-modulates capping at G18 [23] acts on the GTase but does not affect the TPase, because the TPase had to have acted during the pulse when the modulator was present.

**Fig 5:**
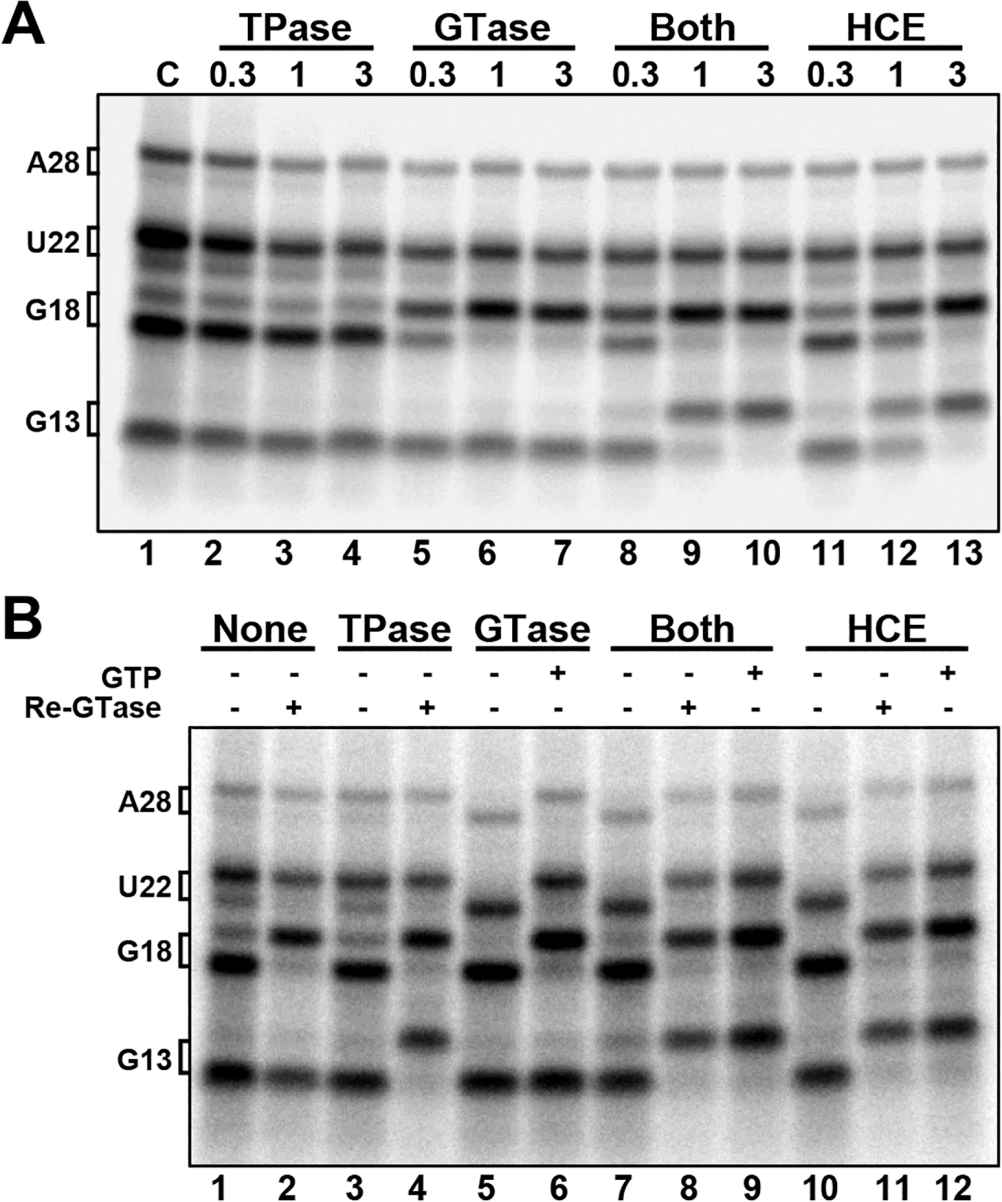
G18 in isolated elongation complexes is a diphosphate. *A*, Addback of TPase, GTase, both TPase and GTase, or HCE to naked RNA (amounts in pmol) *B*, Addback of 3 pmol of indicated factor +/- GTP, +/- reisolation and treatment with GTase and GTP after initial treatment (Re-GTase).

To confirm that uncapped G18 in our HSW EECs is indeed a diphosphate and rule out the possibility that the recombinant GTase was somehow acting on a triphosphate, the nascent RNAs were isolated from the HSW EECs and then incubated with either one or both recombinant domains or HCE (Fig 5A). GTase alone was able to cap G18 but not G13, which is consistent with the HCE triphosphatase domain acting on G18 but not G13 during the pulse (lanes 5–7). This is further supported in lanes 8–10, in which incubation with both the TPase and GTase simultaneously resulted in capping of G13, demonstrating that G13 was not acted upon by the HCE triphosphatase domain during the pulse. These findings explain why in the earlier study [23] we were able to efficiently cap G18 (previously called G21) in HSW EECs.

While it is clear from the data presented thus far that the TPase and GTase can act in tandem, and that the GTase can act completely independently (i.e. in the absence of the TPase), it was not clear if the TPase could act independently of the GTase. To address this, another assay was performed in which TPase, GTase, both, or HCE were incubated with naked RNA; GTP was added only in the indicated lanes, and some indicated lanes had the RNAs reisolated after initial addback and then incubated with GTase and GTP (Fig 5B). This was done to probe for TPase activity, since there is no mobility shift in our gel system that can directly detect TPase activity. Lane 4 shows that TPase can act on G13 in the absence of GTase, since G13 could be capped by GTase after reisolation. Therefore, no allosteric interaction between the two is required for TPase activity. In lanes 7 and 10, in which the RNAs were incubated with either GTase or HCE in the absence of GTP, the RNAs that were capped to begin with lost their cap (compare with the starting material in lane 1). This demonstrates that the human capping reaction is reversible, as has been demonstrated for chlorella virus PBCV1 [31] and vaccinia virus [32] capping enzymes.

## Conclusions

We initially set out to characterize the inhibition of capping that results from H_2_O_2_ treatment because of the possibility that the inhibition could be related to or mediated by the previously described unknown factor that inhibits early capping [23]. As a result of this initial search we determined that H_2_O_2_ inactivates HCE directly through irreversible oxidation of Cys126, the catalytic nucleophile of the triphosphatase domain. This signifies that the loss of capping after H_2_O_2_ treatment of extracts results from direct covalent damage to HCE and is independent of the inhibitor of early capping. This is further supported by the fact that this unknown factor targets the HCE guanylyltransferase activity while not affecting the triphosphatase activity, since addback of GTase to HSW EECs with uncapped G18 leads to complete capping of G18 (Fig 4B). Therefore, the HCE triphosphatase domain acted upon it during the pulse, while the HCE guanylyltransferase domain could not. This also demonstrates that contact with the HCE triphosphatase domain is not required for GTase activity on nascent RNA. So, in contrast to the yeast system [22], the HCE guanylyltransferase domain does not appear to be inhibited from charging by the phosphorylated Pol II CTD. Together these results suggest that the factor that is inhibiting HCE guanylyltransferase activity on G18 during elongation may be modulating that domain’s activity, and that the displacement of this factor as part of the ordered exchange of factors required for productive elongation [23] could facilitate HCE guanylyltransferase activity on the nascent RNA.

Unlike the triphosphatase activity, which differs widely in structure and mechanism between fungi and metazoans, the mechanism and key structural motifs of all known cellular guanylyltransferase enzymes are conserved [33, 34]. Structural studies in *Chollera* virus [35], *C*. *albicans* [36], and other organisms have shown that guanylyltransferase enzymes can exist in either an open or closed conformation. This has led to the proposal of a general mechanism for charging of the enzyme with GTP. The open conformation binds GTP and magnesium, causing a shift to the closed conformation, and this allows GTP-enzyme interactions to occur that catalyze the transfer of GMP to the lysyl-N of the active site lysine, forming the covalent intermediate. Concomitant cleavage of the GTP β-γ phosphates eliminates enzyme-GTP interactions and allows the enzyme to shift back to the open conformation [33]. The enzyme then has to close again to transfer the GMP moiety to diphosphate RNA, and then open to release the capped RNA [37]. This requirement for a cycling between open and closed conformations implies that if the guanylyltransferase were to become locked in one conformation, catalysis would be inhibited. We speculate that the inhibitor of capping at G18 [23] may be regulating the HCE guanylyltransferase domain in this manner. It is worth noting that Ceg1 requires interaction with a specific region of Cet1 (residues 205–265) to become charged [22], demonstrating that allosteric regulation of guanylyltransferase charging is possible.
